# Evaluation of Bacterial Communities of *Listronotus maculicollis* Kirby Reared on Primary and Secondary Host Plants

**DOI:** 10.3390/insects16020114

**Published:** 2025-01-24

**Authors:** Garrett Price, Audrey Simard, Benjamin A. McGraw

**Affiliations:** 1 Department of Entomology, Pennsylvania State University, University Park, PA 16802, USA; gyp5046@psu.edu (G.P.); ajs8275@psu.edu (A.S.); 2 Department of Plant Science, Pennsylvania State University, University Park, PA 16802, USA

**Keywords:** *Agrostis stolonifera*, *Listronotus maculicollis*, microbiota, *Poa annua*, turfgrass

## Abstract

The annual bluegrass weevil (*Listronotus maculicollis* Kirby) is a devastating insect pest of annual bluegrass (*Poa annua* L.) and, to a lesser extent, creeping bentgrass (*Agrostis stolonifera* L.) on golf courses. *Listronotus maculicollis*-reared *A. stolonifera*, a comparatively tolerant host, incurs fitness costs, including longer developmental periods and reduced larval survivorship. This study sought to characterize microbiota diversity in *L. maculicollis* adults and larvae reared on *P. annua* and *A. stolonifera* cultivars (Penncross & A4) to explore whether intrinsic factors, such as microbial community composition, vary across host plants and developmental stages, potentially influencing host suitability. Alpha diversity analyses showed adults feeding on A4 exhibited higher bacterial species richness than their offspring reared on the same cultivar. Beta diversity analysis revealed significant dissimilarities between *L. maculicollis* adults and offspring regardless of host. *Pseudomonas* sp. was consistently abundant in larvae across all turfgrasses, indicating a potential association with larval development. Elevated levels of *Wolbachia* sp., known for insect reproductive manipulation, were observed in adults, but appear to be unrelated to host plant effects. The most prevalent bacterium detected was *Candidatus* Nardonella, a conserved endosymbiont essential for cuticular hardening in weevils. Given the role of cuticular integrity in insecticide resistance, further investigations into insect–microbe–plant interactions could guide the development of targeted pest management strategies, reducing resistance and improving control measures for *L. maculicollis*.

## Simple Summary

The annual bluegrass weevil (*Listronotus maculicollis*) is a serious pest affecting golf courses, mainly damaging annual bluegrass (*Poa annua*) and, to a lesser extent, creeping bentgrass (*Agrostis stolonifera*). Weevils developing with creeping bentgrass develop slower and experience greater mortality rates than weevils within annual bluegrass. This study looked at the different bacterial communities within weevil adults and larvae raised on these two types of grass and cultivars to determine if plant nutrition affects these outcomes. Adult weevils feeding on a newer creeping bentgrass cultivar (A4), had more diverse bacteria than their larvae. Significant differences in the bacteria taxa between adult weevils and larvae were observed regardless of which grass they consumed. *Pseudomonas* sp. was common in larvae and may be linked to their growth and development. *Wolbachia*, a bacterium known to affect insect reproduction, was more common in adults and probably not influenced by their host plant type. The most common bacterium found was *Candidatus* Nardonella, which assists in the rigidity of the weevil’s exoskeleton. A further study of these relationships between the annual bluegrass weevil, host plants, and bacteria associated with exoskeleton hardening may better inform management programs and reduce the impact of insecticide resistance populations.

## 1. Introduction

Microbiota refers to the diverse community of microorganisms inhabiting a specific environment or host, including bacteria, fungi, viruses, and other microbes [1]. In insect systems, these microbial communities are essential to various physiological processes, such as metabolism and detoxification, and to mediating interactions with host plants (antibiosis and antixenosis) and other environmental factors [2,3,4]. Gut microbiota can enable insects to break down toxic plant compounds, thereby expanding their range of viable host plants and potentially contributing to pesticide resistance [5,6]. These endosymbionts are often passed via vertical transmission from mother to offspring, and may be acquired horizontally from the environment (e.g., soil and host plants) [7]. While similar plant species tend to harbor similar core sets of microbes [8], polyphagous insect herbivores can influence their microbiota dependent on the host plant choice [9,10,11].

The annual bluegrass weevil (*Listronotus maculicollis* Kirby) is the most severe insect pest of short-mown (0.6–2 cm) turfgrass typically found on golf courses across eastern North America [12,13,14]. The insect was first detected damaging turfgrass in 1957 on Long Island, NY. Since this time, the distribution of damaging populations has radially expanded outwards to now include states as far south as Georgia, north from Ontario to the Canadian Maritime Provinces, and west to Arkansas, Kansas, and Nebraska [15]. *Listronotus maculicollis* damage is most common in turf stands with a high percentage of annual bluegrass (*Poa annua* L.), particularly in the areas surrounding the epicenter of its distribution (metropolitan New York area). Since the early 2000s, reports of damage to creeping bentgrass have become prevalent during southern range expansion into Mid-Atlantic states. Host tolerance to *L. maculicollis* herbivory varies [16,17]. In stands composed of *P. annua* and *A. stolonifera*, these differences in tolerance give the appearance of selectivity and preference towards *P. annua* [18]. Previous no-choice studies have demonstrated that *A. stolonifera*-reared *L. maculicollis* larvae gain less weight and experience lower survivorship when compared to those reared on *P. annua* [18,19,20].

Traditionally, *L. maculicollis* management has centered around controlling adults as they leave overwintering sites (e.g., leaf litter, tall grasses, and other protected habitats surrounding the short-mown playing surfaces) in spring with broad-spectrum insecticides [21,22]. Once oviposition has occurred, early instars (first–third-instar larvae) are largely protected from most controls as they bore through stems. Fourth- and fifth-instar larvae emerge in the soil, and may be targeted with larvicides. However, these applications are generally more expensive than adulticides and require precise timing, as these stages feed on the apical meristem, which leads to plant death [13]. Considering the insect’s biology, the severity of the damage it inflicts, and the stringent aesthetic standards required on golf courses, it is not surprising that multiple adulticide applications are frequently employed, which inevitably contributeds to pyrethroid resistance in many populations [23], and more recently multiple resistance with unrelated compounds [22,24].

Microbial-mediated metabolism has been proposed to play a pivotal role in the biodegradation of pesticides in the environment, and the detoxification of plant-derived phytotoxins and synthetic insecticides within agricultural systems [1,25]. While the microbiota of some turfgrass pests, such as the clover root weevil (*Sitona obsoletus*, Fab) and Japanese beetle (*Popillia japonica*, Newman), have been characterized, the composition and function of the *L. maculicollis* microbiota remains understudied [26,27]. *Listronotus maculicollis* serves as an ideal model for studying mutualistic interactions with endosymbionts due to its polyphagous nature and variable development on different host plants [18,20]. Numerous studies have demonstrated that weevils harbor bacterial endosymbionts essential to their development and metabolism [28,29,30]. *Listronotus maculicollis* may utilize microbial symbionts to facilitate host plant shifts due to nutrient supplementation and the detoxification of plant defensive compounds when challenged with non-preferred host plants and cultivars [31,32]. The ingestion of phytotoxins may elicit plant defense-induced dysbiosis, resulting in rapid and often deleterious shifts in gut-microbial communities, and can be lethal to susceptible insects [33,34]. Conversely, numerous endosymbionts within coleopteran mid-guts have been associated with detoxifying plant secondary metabolites ingested in plant foliar tissues [35]. The development of pyrethroid- and multiple-resistant *L. maculicollis* populations within a relatively short period of time (<10 years) suggests that the weevil may acquire symbionts from the environment that allow for tolerance or facilitate the detoxification of insecticides.

Despite the critical role of microbiota in various insect systems, the bacterial communities associated with *L. maculicollis* remain largely uncharacterized. Understanding the weevil’s microbiota is essential for revealing potential microbial interactions that may influence host plant utilization and contribute to its adaptation to insecticides and other management practices. The current study sought to determine the following: (1) Does the microbiota diversity and composition of adult and larval weevils vary across turfgrass varieties? (2) What taxa are associated with each turfgrass variety? To characterize the bacterial microbiota associated with *L. maculicollis* adults and larvae on three varieties of turfgrass, creeping bentgrass (*A. stolonifera* c.v. A4 and Penncross) and annual bluegrass (*P. annua*, wildtype), a metabarcoding approach was utilized. It is our hope that the study furthers our understanding of plant–insect–microbe interactions, providing valuable insights into these complex interactions’ ecological and evolutionary dynamics.

## 2. Materials and Methods

### 2.1. Turfgrass Growth and Maintenance

Plants were established from seed (*A. stolonifera* cv. Penncross (PC), *A. stolonifera* cv. A4 (A4), and wild-type *P. annua* (PA)) sown into plastic growth containers (30 cm × 15 cm × 4 cm) and held in a greenhouse throughout the study. Seedlings were grown on a pasteurized mixture of 3:1 (sandy loam soil:top dressing sand), watered as necessary, and clipped twice a week to maintain a consistent height of cut (0.6–2 cm). Plants were maintained as such for ten weeks until use in bioassays.

### 2.2. Insects

*Listronotus maculicollis* adults were collected using a reverse leaf-blower vacuum (Echo ES 255, Echo Inc., Lake Zurich, IL, USA) from short-mown *P. annua* turf located in central Pennsylvania (Joseph E. Valentine Turfgrass Research Center, University Park, PA, USA) on 11 April 2023. Weevils were placed within plastic containers with mesh lids and transported to the Turfgrass Entomology Laboratory at Pennsylvania State University (University Park, PA, USA). Upon arrival, adults were assessed for viability and sexed. Males and females were kept separately in 840 mL plastic containers (groups of up to 500) with mesh lids. Containers were placed into a climate-controlled incubator (14 h light at 21 °C/10 h dark at 14 °C) until use in bioassays.

Adults were infested in a flat tray (50 × 25 cm) confined within a collapsible mesh terrarium containing either *P. annua* or *A. stolonifera* (c.v. Penncross and A4) turf grown on sterilized soil from seed. Each tray (n = 2 per turfgrass type) was infested with 20 males and 20 females, with individual weevils serving as biological replicates. After fourteen days, weevils were manually removed from the arena over three days or until 100% recovery was achieved to standardize larval age and avoid continuous oviposition. Turfgrass sod was removed from flat trays and inverted, and then placed on Berlese funnels (BioQuip Products Inc., Rancho Dominguez, CA, USA) to heat-extract the larvae. Cores were held at 40 °C in a darkened incubator for 48 h. The extracted larvae were counted, and head capsules were measured to determine developmental age (instar) [21]. To standardize the assessment of the larval developmental stage, the larval instar average (L_avg_) was calculated using the following formula [22]:

L_avg_ = [nL1 × 1 + nL2 × 2 + nL3 × 3 + nL4 × 4 + nL5 × 5]/N

where n represents the number of individuals recovered for each instar (L1 through L5 as the five larval stages) and N is the total number of all stages recovered. This approach allowed for a standardized measure of developmental stage, reflecting larval age across samples. Using L_avg_ mitigates the potential variation in developmental timing and ensures consistency in the analysis of bacterial communities, reducing the bias introduced by mixed developmental stages.

### 2.3. DNA Extraction and Library Preparation

Adults (n = 5) and larvae (n = 10) reared on each turf cultivar were randomly selected from the original mating pairs used to infest the trays. Adults were standardized by selecting individuals of similar size and developmental stage, and collected simultaneously to minimize variability. Equal numbers of males and females were included to balance potential gender-related differences. To control for other variables that could influence endomicrobial presence, all individuals were reared under identical conditions within a climate-controlled setup. Selected adults and larvae were surface-sterilized with 10% NaOCl, followed by 95% EtOH, and rinsed with sterile, deionized H_2_O. Total DNA was extracted from surface-sterilized adults using a DNeasy Blood & Tissue Kit (QIAGEN, Hilden, Germany) using the manufacturer’s protocol for the purification of total DNA from insects. Insects were homogenized via disposable mortar and pestle using a 10% phosphate buffer solution, and final elution was performed twice with 100 µL of Buffer Alkaline Elution (AE). 16S rRNA genes from the hypervariable region V4 were amplified using GoTaq Green Master Mix (Promega; Madison, WI, USA) and the following primers:

- 515F-v1 (GTGYCAGCMGCCGCGGTAA);
- 806R-v1 (GGACTACNVGGGTWTCTAAT).

These primers were previously used by the Earth microbiome project [36,37] and were provided by the Huck Genomics Core at Pennsylvania State University upon request. The lengths of the resulting amplicons were verified via gel electrophoresis, purified using a Wizard SV Gel and PCR Clean-Up System (Promega; Madison, WI, USA) and purity-checked via a Nanodrop 2000c (ThermoScientific; Wilmington, DE, USA), and their concentrations were checked via a Qubit fluorometer (Thermo Fisher; Waltham, MA, USA). Samples were submitted to the Huck Genomics Core Facility at Pennsylvania State University for a second round of PCR to add the indexes and Illumina adapters to the amplicons to create a library. Amplicons were combined into an equimolar pool and sequenced using MiSeq 250 × 250 paired-end sequencing.

### 2.4. Metagenomic Analysis

Bioinformatic analysis was conducted using Mothur v. 1.48.0 [38], following the MiSeq SOP with minor modifications. Raw FASTQ files were downloaded from the Huck Genomics Core Facility server and converted to FASTA format using the make.contigs command. Quality control steps included combining reads into contigs, removing sequences with ambiguous bases, filtering out sequences longer than 275 base pairs, and aligning sequences to the SILVA database v138.1, trimmed to the V4 region using the align.seqs command. Chimeras were identified and removed using the chimera.vsearch command. Sequence redundancy was reduced using the unique.seqs and pre.cluster commands to enhance data quality. Operational taxonomic units (OTUs) were clustered using the cluster.split command, and consensus taxonomy was assigned to each OTU using the classify.otu command. Standardization was achieved by subsampling all libraries to the size of the smallest library (1286 sequences), ensuring consistent comparisons across datasets.

Alpha diversity metrics, including observed OTUs, sequence coverage, and the inverse Simpson diversity index, were calculated using the summary.single command with subsampling. Beta diversity was assessed using the Bray–Curtis dissimilarity index (dist.shared command), and the resulting distance matrices were visualized using non-metric multidimensional scaling (NMDS) plots generated in R.

Statistical analyses included the analysis of molecular variance (AMOVA) to evaluate whether the genetic diversity of microbial communities differed significantly among the three turf cultivar populations. The model used population identity (turf cultivar) as the main grouping variable, and pairwise distance matrices (e.g., Bray–Curtis) as the response variable, comparing within-group versus between-group variance. The homogeneity of molecular variance (HOMOVA) was used to determine whether the variation between populations was significantly different. The OTUs responsible for shifting samples along NMDS axes were identified by calculating Pearson’s correlation coefficients between the relative abundance of each OTU and the axes using the corr.axes command in Mothur, highlighting OTUs most strongly associated with the microbial community structure.

Differentially abundant OTUs were identified using Metastats, and representative sequences for OTUs were generated using the get.oturep command. These sequences were then searched using NCBI’s BLAST tool to identify OTUs, focusing on those with greater than 1% relative abundance in any sample. This comprehensive analysis ensured high-quality data and robust statistical evaluations of microbial community differences.

## 3. Results

Forty-five samples (n = 5 adults and 10 larvae per cultivar) produced 863,803 reads for downstream analysis. Sequences were assigned to 1579 unique OTUs, with 837 OTUs having only one representative and the top 10 OTUs comprising >86% of sequences. Samples were rarefied at the lowest library size (1286), resulting in >97% coverage across all samples, indicating that the microbial communities were sufficiently sampled.

**Figure 1:**
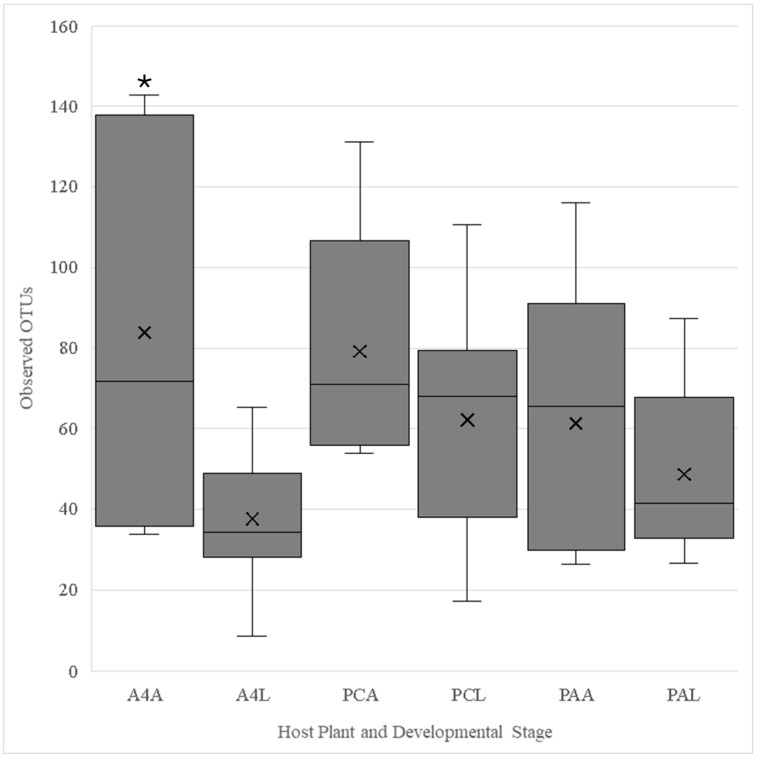
Observed OTUs of the V4 region of 16S rRNA extracted from *L. maculicollis* adult (**A) and larval (**L) *Listronotus maculicollis* collected from *Agrostis stolonifera* (A4* and PC*) and *Poa annua* (PA*). Columns denoted by “*” indicate significant differences in observed OTU richness between L. maculicollis development stages per turfgrass cultivar (α = 0.05). Boxes cover the interquartile interval where 50% of the data are found, with the line splitting the boxes indicating the median. Whiskers indicate the values furthest from the center while still within 1.5 times the interquartile range. Xs indicate the means of the data.

**Figure 2:**
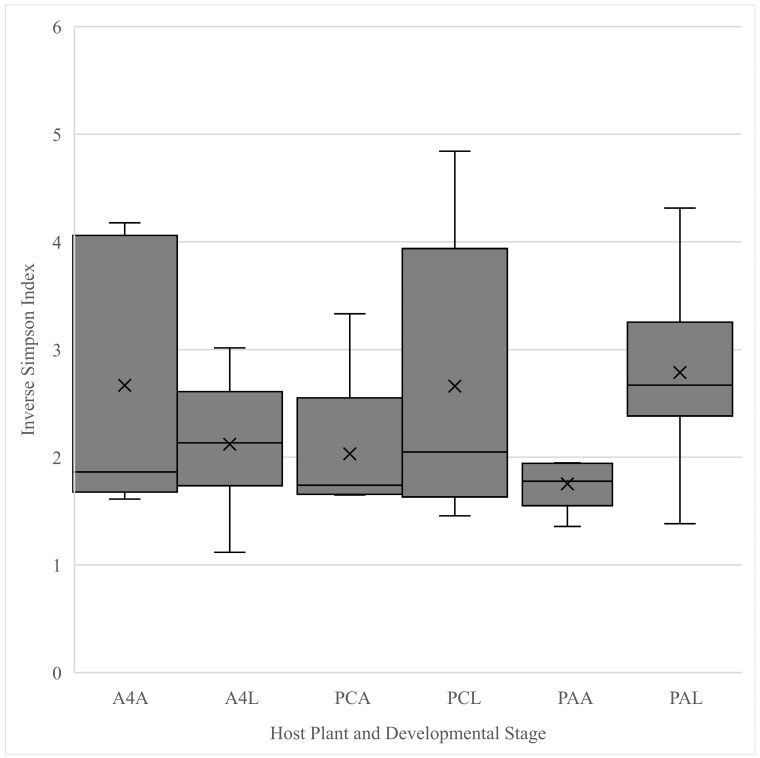
Box and whisker plot of the Inverse Simpson alpha diversity of OTUs determined from sequences of the V4 region of 16S rRNA from adult (**A) and larval (**L) *Listronotus maculicollis* collected from *Agrostis stolonifera* (A4* and PC*) and *Poa annua* (PA*). Boxes cover the interquartile interval where 50% of the data are found, with the line splitting the boxes indicating the median. Whiskers indicate the values furthest from the center while still within 1.5 times the interquartile range. Xs indicate the means of the data.

Adults reared on A4 had significantly more observed OTUs than their offspring (*F*_1,13_ = 7.13, *p* = 0.02) (Figure 1), but no significant differences in diversity were detected (*F*_1,13_ = 1.30, *p* =0.27) (Figure 2). No significant differences in the numbers of observed OTUs were found between PA-fed adults and their offspring (*F*_1,13_ = 0.74, *p* = 0.41) (Figure 1). However larval microbiota was significantly more diverse (*F*_1,13_ = 7.33, *p* = 0.02) (Figure 2). There was no significant difference in either the number of observed OTUs (*F*_1,13_ = 1.18, *p* = 0.30) (Figure 1) or the diversity (*F*_1,13_ = 1.09, *p* = 0.32) (Figure 2) between the adults and larvae fed on PC. Comparisons of the adults fed on *P. annua*, *A. stolonifera* c.v. Penncross and A4 showed no significant differences in observed OTUs (*F*_2,12_ = 0.43, *p* = 0.66) (Figure 1) or diversity (*F*_2,12_ = 1.48, *p* = 0.27) (Figure 2). Similarly, the larvae reared on different turfgrasses showed no significant differences in observed OTUs (*F*_2,27_ = 3.02, *p* = 0.07) (Figure 1) or diversity (*F*_2,27_ = 1.46, *p* = 0.25) (Figure 2).

**Figure 3:**
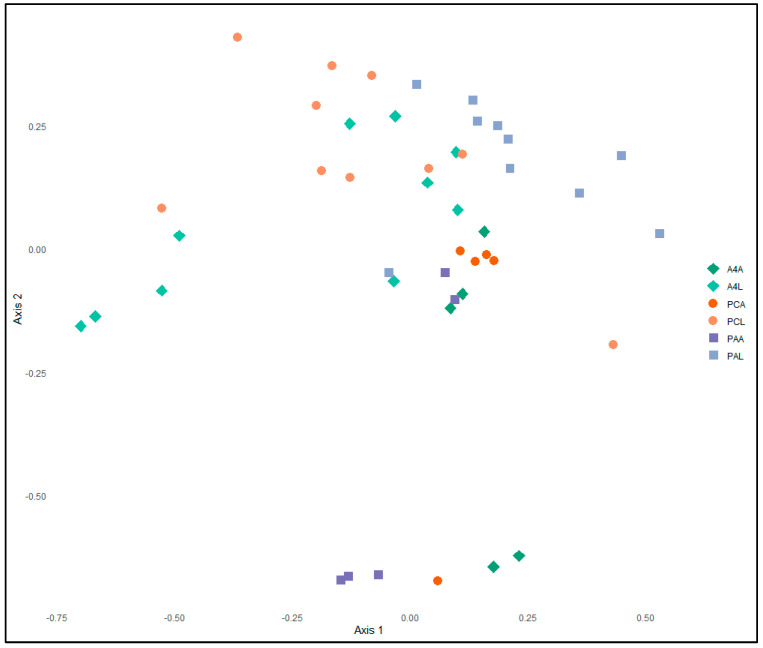
Non-metric multidimensional scaling (NMDS) plot of the first two dimensions of OTUs based on Illumina MiSeq sequencing of the V4 region of the microbial 16S rRNA gene from adult (**A) and larval (**L) *Listronotus maculicollis* collected from *Agrostis stolonifera* (A4* and PC*) and *Poa annua* (PA*). The distance between points is relative to the distance between samples. Analysis of molecular variance confirmed that the observed separation between populations is statistically significant (*p* < 0.001).

NMDS analysis performed on the distance matrix of the samples in three dimensions, utilizing the Bray–Curtis distance measure, yielded a stress value of 0.12, suggesting an acceptable fit of the ordination to the distance matrix. The ordination model explained a significant proportion of the variability in the dissimilarity matrix, as evidenced by an R-squared value of 0.92 (Figure 3). Significant differences were detected between *L. maculicollis* bacterial communities between primary (*P. annua*) and secondary host plants (*A. stolonifera* c.v. Penncross and A4) (*F*_5,39_ = 5.21, *p* < 0.001), indicating that the microbiota composition is distinct and influenced by the host plant type. Specific comparisons showed significant differences between the adults and their offspring for insects fed on all varieties (A4: *F*_1,13_ = 5.13, *p* < 0.01; PC: *F*_1,13_ = 3.55, *p* = 0.05; PA: *F*_1,13_ = 12.18, *p* = 0.001). Pairwise comparisons of the adult microbiotas via Metastats showed significant differences between the PC- and the PA-fed adults (*F*_1,8_ = 2.51, *p* = 0.05), but no significant differences between the bentgrass-fed adults (*F*_1,8_ = 0.88, *p* = 0.20) or between A4- and PA-adults (*F*_1,8_ = 1.07, *p* = 0.24). The microbiotas of the PA-reared larvae and larvae reared on either bentgrass cultivar were significantly different from each other (A4: *F*_1,18_ = 6.46, *p* < 0.001; PC: *F*_1,18_ = 3.88, *p* = 0.01). Conversely, there was no significant difference between the microbiotas of the larvae reared on the two bentgrass cultivars (*F*_1,18_ = 1.89, *p* = 0.128). The HOMOVA showed no significant differences in the variation between treatments (*p* = 0.129).

**Figure 4:**
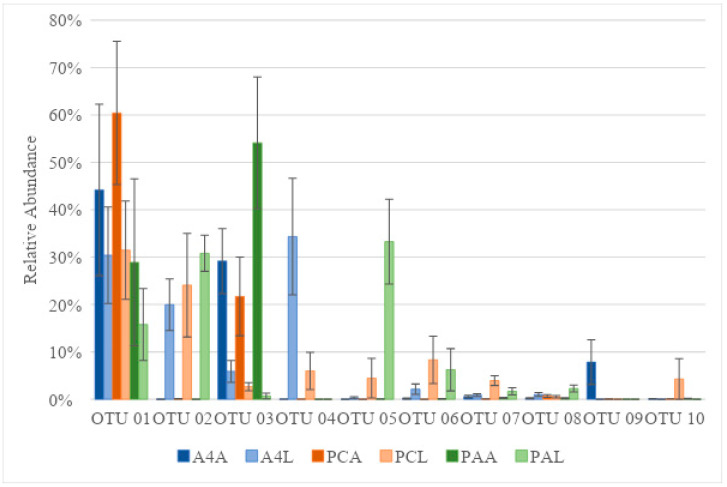
Relative abundances of the first ten most abundant OTUs based on Illumina MiSeq sequencing of the V4 region of the microbial 16S rRNA gene from adult (**A) and larval (**L) *Listronotus maculicollis* collected from *Agrostis stolonifera* (A4* and PC*) and *Poa annua* (PA*).

Ten OTUs represented 86% of all sequences observed, each exhibiting greater than 4% relative abundance in treatments (Table 1; Figure 4). The Metastats comparison of the microbiota of the adults and their offspring showed a significantly elevated relative abundance of OTU 2 (*Pseudomonas* sp.) (A4, *p* < 0.001; PC, *p* = 0.03; PA, *p* < 0.0001) and a significantly reduced abundance of OTU 3 (*Wolbachia* sp.) (A4, *p* < 0.01; PC, *p* = 0.02; PA, *p* < 0.001). Additionally, OTU 4 (*Aeromonas* sp.) was the most abundant in A4-fed larvae (34.3%), which was found in significantly greater levels than in the adults (*p* < 0.01). OTU 5 (*Klebsiella* sp.) was the most abundant in PA-fed larvae (33.3%), and its level was significantly higher than what was detected in adults (*p* < 0.001). Microbiota differences in the larvae reared on different cultivars of bentgrass were primarily driven by OTU 4 (*Aeromonas* sp.), which was significantly elevated in larvae reared on A4 (*p* = 0.04). Adults fed on the different bentgrass cultivars differed only in the relative abundance of OTU 7 (*Sphingomonas* sp.), which was significantly elevated in those reared on A4 (*p* = 0.05). In comparisons of larvae reared on PA and larvae reared on bentgrass, OTU 5 (*Klebsiella* sp.) was significantly elevated in PA larvae (A4, *p* = 0.001; PC, *p* = 0.02) and OTU 5 (*Klebsiella* sp.) was significantly elevated in bentgrass larvae (A4, *p* = 0.001; PC, *p* < 0.01). The microbiotas of the adults fed on PA versus those fed on bentgrass were largely similar, except for OTU 3 (*Wolbachia* sp.), which was significantly elevated in PA-fed weevils over PC-fed weevils (*p* = 0.04).

## 4. Discussion

This study is the first to characterize the bacterial microbiota of *L. maculicollis* and examine how primary and secondary host plants influence microbial community composition across life stages. Microbiota richness and diversity differ significantly between *L. maculicollis* adults and larvae reared on primary (*P. annua*) and secondary (*A. stolonifera*) host plants. Across all life stages and turfgrass cultivars, the ten most abundant OTUs accounted for over 86% of observed sequences, indicating that a few taxa dominated the bacterial microbiota. Adults fed *A. stolonifera* cv. Penncross, an older cultivar compared to A4, also had different microbiotas compared to those fed *P. annua*, with distinct OTU abundance patterns. *Pseudomonas* sp. was more common in larvae across groups, while *Wolbachia* sp. was less prevalent in larvae than in adults. Alpha diversity analysis showed that adults fed *A. stolonifera* cv. A4 had significantly higher bacterial richness than their larvae reared on the same cultivar. *Listronotus maculicollis* larvae reared on *P. annua* exhibited greater microbial diversity, but no significant differences were observed in richness. Beta diversity analysis revealed substantial dissimilarities between adults and larvae across all cultivars. These findings suggest that the *L. maculicollis* microbial community is dynamic between life stages and influenced by their host plant.

The most abundant OTU across all samples, comprising 35.2% of all sequences (OTU 1), was identified as an unknown bacterium. A BLAST search revealed that OTU 1 shared 98.42% identity with an endosymbiotic bacterium from the Argentine stem weevil, *Listronotus bonariensis* Kuschel (Coleoptera: Curculionidae) (GenBank accession no. KJ522448.1) [26]. Both *L. bonariensis* and *L. maculicollis* have similar life cycles and are significant turfgrass pests, damaging plants by feeding on stems and crowns as larvae. In *L. bonariensis*, 89% of sequences correspond to a single bacterium from the family Enterobacteriaceae, associated with *Candidatus* Nardonella [26]. *Candidatus* Nardonella is a group of ancient endosymbionts found exclusively in weevils and thought to have co-evolved with them for over 125 million years [39,40]. These bacteria are believed to play essential roles in the growth and development in Curculionidae, though their functions and mechanisms vary by species and population. For the West Indian sweet potato weevil, *Euscepes postfasciatus* Fairmaire, Nardonella is not essential for reproduction, but has been found to influence body weight, pigment, and growth rate [41]. In *Pachyrhynchus infernalis* Fairmaire, Nardonella synthesizes tyrosine, a critical amino acid for cuticle sclerotization [29]. The high relative abundance and conserved presence of this bacterium in all *L. maculicollis* samples suggest that Nardonella plays a similarly significant role in the life cycle and development of *L. maculicollis*, potentially supporting crucial biological functions regardless of host–plant interactions.

Our analysis has shown that *Pseudomonas* sp. was consistently abundant in larvae amongst all the assessed hosts, indicating a potentially beneficial role in larval development or stress tolerance. *Pseudomonas* species are Gram-negative rods commonly found in diverse environments, including the midgut of insect larvae, where they engage in varied interactions with their hosts [42]. The specific role of *Pseudomonas* in insect larvae can depend on both the bacterial strain and the host species [43,44,45]. Some strains are entomopathogenic, causing disease and death in larvae. For instance, *Pseudomonas taiwanensis* produces an insecticidal toxin that damages the midgut epithelium, leading to septicemia in several agricultural pests [43]. In contrast, other *Pseudomonas* species benefit their host. *Pseudomonas aeruginosa* colonizes the gut of housefly larvae, where it produces antifungal compounds that inhibit the growth and germination of *Beauveria bassiana* spores, thereby protecting larvae from fungal infections [44]. Additionally, *Pseudomonas* spp., associated with bark beetles, can degrade plant defensive compounds like α-pinene, facilitating fungal symbiont growth and aiding beetles in overcoming host tree defenses [45]. To determine the exact role of *Pseudomonas* in *L. maculicollis* larvae, further studies are needed to isolate and identify the specific strain present, followed by knockdown and reinoculation experiments to assess its effects on *L. maculicollis* larval fitness and survivorship.

The third most abundant bacterial taxa (OTU 3), identified as *Wolbachia* sp., showed a significantly higher relative abundance in adult weevils across all turfgrass types. *Wolbachia* is a naturally occurring bacterial endosymbiont that infects many insect species, influencing their reproduction, development, and immunity [46]. Primarily transmitted vertically from mother to offspring through the egg cytoplasm, *Wolbachia* manipulates insect reproductive biology in several ways, including inducing cytoplasmic incompatibility, feminization, male killing, or parthenogenesis [47]. Such manipulations can increase the prevalence of *Wolbachia* within insect populations and reduce host genetic diversity. In addition to reproductive parasitism, *Wolbachia* can provide beneficial functions, such as supplying essential nutrients, enhancing pathogen or parasite resistance, and increasing thermal tolerance [48,49,50]. Its abundance within insect hosts can vary with tissue type, mode of transmission, and developmental stage [51]. In the yellow fever mosquito, *Aedes aegypti* L., *Wolbachia* is more prevalent in adult females’ reproductive tissues than other tissues or larvae [52]. Wolbachia has been identified in 204 beetle species, with an average prevalence of 38.3%, varying significantly across families and genera, and causing various effects such as cytoplasmic incompatibility, male-killing, and selective sweeps [53]. Phylogenetic analyses reject cospeciation between Wolbachia and beetles, instead supporting horizontal transmission, particularly among beetles sharing habitats or host plants [53,54]. The high relative abundance of *Wolbachia* in adult *L. maculicollis* suggests a role in reproductive manipulation or mutualism, potentially influencing reproductive success and immunity, as documented in other weevil species [30].

Beta diversity analysis showed significant differences in microbiota composition across life stages and turf cultivars. Pairwise comparisons revealed notable distinctions between adults and larvae and between *A. stolonifera* cv. Penncross and *P. annua*. Insects can acquire their microbiota vertically, from parent to offspring, or horizontally, from the environment or other sources [55]. Vertical transmission typically leads to high similarity in microbiota between adults and their offspring, as shared bacterial strains adapt to the insect host and diet [56]. Horizontal transmission, however, can result in more significant differences in microbiota between adults and offspring, as insects acquire bacterial strains that vary in abundance and function depending on available microbial substrates and competition [57]. However, transmission modes are not always strictly vertical or horizontal. Some insects employ a mixed mode of transmission, inheriting certain bacteria from their parents while acquiring others from the environment. This mixed mode of transmission may account for bacteria that are consistently abundant across all life stages of *L. maculicollis* and host plants and those that vary between specific treatments. Future research should focus on identifying the functional roles of endosymbionts, particularly *Pseudomonas* and *Wolbachia*, in *L. maculicollis* physiology and adaptation. We can further our understanding of microbial–insect–plant interactions and host–plant suitability by investigating microbiota variations in insect pests reared on different hosts.

Host plants affect insect physiology, nutrition, immunity, and microbial communities [57,58]. The diversity and richness of insect-associated bacteria vary between primary and secondary host plants. Transitioning from a primary to a secondary host plant can increase bacterial diversity, introducing more specialized taxa to better adapt to novel plant compounds or combat commensal pathogens [58,59]. Conversely, returning to a primary host may reduce bacterial diversity as certain microbes become redundant [58]. The differences observed between *A. stolonifera* cv. A4-fed adults and *P. annua*-fed larvae likely reflect each host plant’s specific nutritional and biochemical compositions, which may necessitate specialized bacteria to process unique plant compounds. Shifts in insect microbiota depend on factors such as the duration of host plant exposure, the insect’s level of specialization on specific plant species or cultivars, and the stability and resilience of the existing microbiota [34,57]. *Poa annua* is uncommercialized and requires extensive management, including frequent mowing, plant growth regulation, fertilization, and pesticide applications, to maintain high-quality turf on valuable playing surfaces. Due to its invasive nature, golf course superintendents managing *A. stolonifera* often view *P. annua* as an undesirable weed [59]. *Agrostis stolonifera* cv. Penncross and the “A Series” (A1–4) were selectively bred in 1954 and the early 1980s to improve turf quality, respectively. Penncross was designed to withstand lower mowing heights and finer texture, while the A-series offers enhanced density, disease resistance, and heat tolerance [60,61]. Significant differences in microbiota composition across host plants emphasize the impacts of host-specific factors in shaping these communities and enhancing *L. maculicollis*’s ability to feed and develop within tolerant cool-season turfgrass, despite fitness costs.

Our investigation into *L. maculicollis* microbiota demonstrated the dynamic interactions of bacteria communities across life stages and host plants. The prevalence of specific OTUs, such as *Pseudomonas* sp. and *Wolbachia* sp., indicates potential ecological interactions that merit further exploration. Conserved bacterial taxa, like *Wolbachia* and *Candidatus* Nardonella, could be targeted for microbial disruption, potentially in conjunction with traditional control methods. This approach could provide novel alternative management strategies for *L. maculicollis*, particularly pyrethroid-resistant populations.

## Figures and Tables

**Table 1:** The 10 most abundant operational taxonomic units (OTU) and their classifications based on a reference alignment, classification based on a BLAST search of the representative sequence, and the relative abundance of each OTU in adult (**A) and larval (**L) *Listronotus maculicollis* collected from *Agrostis stolonifera* (A4* and PC*) or *Poa annua* (PA*).

OTU	Alignment Classification	Top BLAST Result	BLAST Coverage	BLAST % Identity	Relative Abundance						
					A4A	A4L	PCA	PCL	PAA	PAL	All
1	Unknown Bacteria	*L. bonariensis* endosymbiont	100%	98.42%	44.20%	30.40%	60.40%	31.50%	28.90%	15.80%	35.20%
2	*Pseudomonas* sp.	*Pseudomonas* sp.	100%	100.00%	0.00%	20.00%	0.10%	24.10%	0.00%	30.80%	12.50%
3	Unknown Bacteria	*Wolbachia* sp.	100%	100.00%	29.20%	5.90%	21.70%	2.70%	54.10%	0.70%	19.00%
4	*Aeromonas* sp.	*Aeromonas* sp.	100%	100.00%	0.00%	34.30%	0.00%	6.00%	0.00%	0.00%	6.70%
5	*Klebsiella* sp.	Unknown Enterobacteriaceae	100%	100.00%	0.00%	0.30%	0.00%	4.50%	0.00%	33.30%	6.30%
6	*Pseudomonas* sp.	*Pseudomonas* sp.	100%	100.00%	0.20%	2.10%	0.00%	8.30%	0.10%	6.20%	2.80%
7	*Sphingomonas* sp.	*Sphingomonas* sp.	100%	100.00%	0.60%	0.90%	0.10%	4.00%	0.30%	1.70%	1.20%
8	*Pseudoxanthomonas* sp.	*Pseudoxanthomonas* sp.	100%	100.00%	0.20%	1.00%	0.70%	0.50%	0.20%	2.30%	0.80%
9	*Chryseobacterium* sp.	*Chryseobacterium* sp.	100%	100.00%	7.90%	0.00%	0.00%	0.00%	0.00%	0.00%	1.30%
10	*Enterococcus* sp.	*Enterococcus* sp.	100%	100.00%	0.10%	0.00%	0.10%	4.30%	0.10%	0.00%	0.80%

## Data Availability

Data are contained within the article.
